# Patient experience during medical visits in predominately African American rural communities in Alabama

**DOI:** 10.3389/frhs.2025.1420698

**Published:** 2025-06-11

**Authors:** Felecia Wood, Lisle Hites, Pamela Payne-Foster, Morgan M. Newman, Sharlene D. Newman

**Affiliations:** ^1^ Capstone College of Nursing, University of Alabama, Tuscaloosa, AL, United States; ^2^ Department of Population Health, College of Community Health Sciences, University of Alabama, Tuscaloosa, AL, United States; ^3^ School of Architecture, Carnegie Mellon University, Pittsburgh, PA, United States; ^4^ Department of Psychology, Alabama Life Research Institute, University of Alabama, Tuscaloosa, AL, United States

**Keywords:** patient experience, clinician-patient communication, age, gender, African American

## Abstract

Patient experience, specifically, shared decision making, has been demonstrated to significantly affect patient outcomes. The current study examined the clinician-patient communication (CPC) experiences of residents in predominately rural African American communities in Alabama. The 255 participants completed a survey designed to assess aspects of patient experience at their last clinical visit. A third of participants reported not being satisfied with their most recent clinical visit; a majority (55.7%) of participants reported their clinician did not ask their opinion. Participants over age 65 reported greater patient satisfaction and respect compared to younger participants aged 18–45 years. Trending gender effects showed that females reported being listened to more and were more likely to report being treated with respect than male participants. The results of the study show that the clinician-patient experience in rural predominately African American communities represents an opportunity to improve health care outcomes and minimize racial disparities.

## Introduction

Alabama has historically ranked near the bottom in most health metrics. For example, the 2022 *America's Health Rankings* by the United Health Foundation placed Alabama 46th in overall health and 47th in health outcomes (1). These poor rankings are compounded by significant racial disparities. Alabama's diabetes rate stands at 15.1%, compared to the national average of 10.9%, and African American adults in the state face an even higher rate of 18.9% (2). Similarly, hypertension affects 42.7% of Alabamians—10 percentage points above the national average of 32.4%—with African American residents experiencing a rate of 48.4% (3). Additionally, Alabama has the second-highest stroke mortality rate in the U.S. (4). These disparities contribute to increased rates of physical disability and premature death.

Multiple structural and interpersonal barriers fuel these disparities, especially in rural African American communities. Limited access to primary care (5) often forces individuals to miss work for medical appointments, further reducing income and adding financial stress. Beyond access issues, a growing body of research links patient experience to health outcomes (6, 7). Delayed preventive care complicates the management of chronic illnesses like diabetes and hypertension, often leading to worse outcomes (8). Interpersonal barriers—such as communication breakdowns, perceived disrespect, and lack of involvement in care decisions—can further discourage care-seeking, especially among rural residents (9–11). When financial strain, travel burdens, and a lack of trust in providers combine, patients may feel that pursuing preventive care is not worth the effort, further worsening health outcomes.

Patient-centered care offers a potential solution. This model prioritizes the patient's experience, treating individuals as unique partners in their health journey (12). According to Stewart (13), patient-centered care involves: (1) exploring the patient's reasons for the visit; (2) understanding the patient's life context; (3) building shared understanding of problems and treatments; (4) promoting prevention and wellness; and (5) maintaining an ongoing relationship between patient and clinician. This approach aligns with the *Crossing the Quality Chasm* report by the National Academy of Medicine, which promotes shared decision-making (SDM) as a standard of care (14). In SDM, patients collaborate with providers and are full partners in decision-making (15).

At the heart of SDM and patient-centered care is high-quality clinician-patient communication (CPC) which allows for a trusting relationship. The clinician-patient relationship significantly influences whether low-income and minority patients attend clinical visits (16–18). Effective CPC—marked by patient engagement and information sharing—has been linked to better information retention, adherence to treatment, and improved health outcomes (19–21). Unfortunately, studies show that racial disparities often manifest in these interactions. For example, Johnson et al. (19) found that clinicians were more verbally dominant and less patient-centered with African American patients than with White patients, also showing less positive affect. Additionally, Chapman et al. (16) interviewed low-income residents in rural Western U.S. communities and found that the clinician-patient relationship including agreement with treatment plans and positive interactions, affect their interest in attending a clinic visit.

There remains a significant gap in research on patient experience in rural, low-income, predominantly African American communities, particularly in the Deep South. Interestingly, the 2019 Consumer Assessment of Healthcare Providers and Systems survey reported that the Southern U.S. received higher provider communication scores—such as listening carefully, explaining clearly, showing respect, and spending adequate time with patients—compared to other regions (22). Based on existing literature, these scores should correlate with improved health outcomes (23). However, many Southern states, including Alabama, continue to exhibit some of the worst health outcomes in the country.

The goal of the current study was to examine the patient experience in rural, low-income, predominantly African American communities in Alabama.

## Methods

### Participants

A convenience sample of 255 individuals who lived in rural, predominately African American communities in Alabama participated in the study. Most (67%) were female; 83% were African American. Many of the participants were 18–45 years old (113; 44%); 31% ranged from 46 to 65 and the remaining 70 (27%) were older than 65. The study was approved by the University of Alabama Institutional Review Board.

### Participant recruitment

Participants were recruited at community events including health fairs, community festivals, church events, etc. Members of the study team described the survey to participants and those agreeing to participate completed the survey via paper and pencil. No compensation was provided. The communities targeted included five small rural Alabama towns with large African American populations (see Supplementary Materials for descriptions of the towns). Rural was defined using the US Census Bureau's definition (24)—any population, housing, or territory NOT in an urban area. These communities were participating in a health literacy project (25) and had community workers trained to administer surveys and who had completed human ethics training. Efforts were made to recruit a diverse sample within the target communities.

### Survey

The survey used was developed by the investigators and was designed to assess patient experience at their most recent medical visit. Survey items were taken from validated patient experience surveys including the CAHPS Clinician and Group Adult Survey (22, 26) and the Picker Patient Experience Questionnaire (27). The survey consisted of demographic information as well as simple questions to assess aspects of the patient experience. Questions included multiple choice questions: (1) How would you rate the friendliness/responsiveness of the staff? (2) How long did you have to wait for your appointment to start? (3) Did the care providers ask for your opinions? (4) Did the care providers listen to what you had to say? (5) Did the care providers treat you with respect? (6) Was your privacy respected and maintained? (7) When you asked questions, did you get answers you could understand? and (8) How satisfied were you with your visit? Most of the questions had five response options with either a “neutral” mid-point or “about half the time” mid-point.

### Analysis

A logistic regression was performed using SAS 9.4 (28). The dependent variables were questions from the survey. The responses were dichotomized (e.g., Most of the time/Always were combined; Somewhat satisfied/Extremely satisfied were combined; remaining three response options were combined). The independent variables were age (18–45, 46–65, and 65+ which was the comparison) and gender (Male was the comparison). A Spearman Rank correlation was also performed on the full range of ordinal responses.

## Results

### Satisfied with visit

64.3% of participants reported being somewhat or extremely satisfied with their most recent visit. The correlation analysis showed that visit satisfaction was significantly correlated with age (*p* < 0.001; older participants were more satisfied with the visit. The logistic regression analysis showed that those over the age of 65 had significantly higher visit satisfaction than the 18–45 age group [*β* = 0.43, SE = 0.18, Wald = 5.84, *p* = 0.016, 95%CI (1.19, 4.48]; there was no significant difference between 65+ and 46–65. There were no effects of gender or an interaction between gender and age.

### Provider asked opinion

44.3% of participants reported that their clinician asked for their opinion most of the time or always. There were no significant effects of gender or age.

### Provider listened

62.7% of participants reported that their clinician listened to them most of the time or always. The correlation analysis did not reveal an association between being listened to and age. The logistic regression failed to show effects of age when comparing the over age 65 participants to the 18–45 and 46–65 age groups. There was a trend for gender with female participants reporting being listened to more than male participants [*β* = 0.26, SE = 0.14, Wald = 3.42, *p* = 0.064, 95%CI (0.97, 2.86)].

### Provider treated with respect

72.9% of participants reported that their clinician treated them with respect most of the time or always. The correlation analysis showed that respect was significantly correlated with age (*p* = 0.014; older participants felt more respected). The logistic regression showed a trending effect of age when comparing the over 65 age to the 18–45 age group [*β* = 0.36, SE = 0.19, Wald = 3.58, *p* = 0.059, 95% CI (0.36, 1.4)]; there was no significant difference between 65+ and 46–65. There was a trend for gender with female participants reporting being respected more than male participants [*β* = 0.29, SE = 0.15, Wald = 3.67, *p* = 0.055, 95% CI (0.99, 3.17)].

### Provider respected privacy

73.7% of participants reported that their clinician respected their privacy most of the time or always. The correlation analysis showed that respect for privacy was marginally significantly correlated with age (*p* = 0.059; older participants reported their privacy was respected more than younger adults). The logistic regression analysis showed that the over 65 age group had significantly higher reported privacy than the 18–45 age group [*β* = 0.5, SE = 0.19, Wald = 6.63, *p* = 0.01, 95% CI (0.26, 1.06)]; there was no significant difference between 65+ and 46–65. There were no significant gender effects.

### Provider answered questions

Many (67.8%) participants reported that their clinician answered their questions most of the time or always. There were no significant effects of gender or age.

## Discussion

The primary aim of this study was to examine the patient experience among residents of rural, predominantly African American communities in Alabama. Results indicated that patient experience in this population was significantly lower than national benchmarks. For example, only 72.9% of participants in the current study reported feeling they were always treated with respect by their provider, compared to 91% in the 2019 Consumer Assessment of Healthcare Providers and Systems (CAHPS) survey for the South region. Similarly, just 62.7% of participants reported always being listened to by their provider, vs. 88% reported in the CAHPS data.

Notably, more participants felt respected by their provider than felt listened to. One-third (35.7%) reported dissatisfaction with their most recent clinical visit, and over half (55.7%) indicated that their clinician did not ask for their opinion. Age-related effects were observed, with older adults reporting higher satisfaction, greater respect, and more respect for privacy than younger participants. Gender trends emerged as well, with female patients more likely to report being listened to and treated with respect than male patients.

The low percentage (62.7%) of participants who felt listened to is concerning. Given the central role of effective clinician-patient communication in patient-centered care, these findings suggest significant gaps in care quality—particularly among male participants. Previous research has suggested that men are less familiar with the healthcare system due to lower utilization rates, both for themselves and as caregivers, which may impact their comfort level and health literacy (29). Furthermore, Connell et al. (30) noted that cultural norms surrounding masculinity often discourage healthcare-seeking behavior in men, leading to delayed care and reduced self-advocacy. These trends may be even more pronounced in African American men, who are reported to seek care infrequently (31).

Age also influenced perceptions of respect and satisfaction. Younger patients, especially younger women, felt less respected than older patients. DeVoe et al. (32) similarly found that adults aged 18–64 were less likely to report feeling respected or that their provider spent sufficient time with them. These findings may reflect differing expectations—older adults may have more realistic or tolerant views of healthcare interactions, while younger adults may feel their expectations are unmet (33).

Interestingly, respect was reported at higher rates than being listened to. Respect may be influenced by multiple factors, including staff friendliness and wait times. As Cuevas et al. (34) reported, long wait times may trigger feelings of discrimination, impacting perceived respect and satisfaction. Future studies should explore how patients define respect and what they expect from clinician listening behaviors.

### Communication, health literacy, and self-advocacy

Miscommunication in healthcare often stems from differences in verbal, non-verbal, and written communication. Health literacy, the ability to access, understand, and use health information, is critical (35). Universal health literacy precautions, such as using plain language for all patients regardless of background, are essential (36).

A vital part of health literacy is self-advocacy, defined as representing one's interests in healthcare decisions (37). Studies show that self-advocacy improves satisfaction and health outcomes (38, 39). However, disparities exist. Older adults tend to be more passive during medical visits (40, 41), and older African Americans may be even less likely to advocate for themselves due to fears of provider retaliation (38, 43). This may be particularly true in Alabama where there is a history of medical maltreatment of African Americans (e.g., the U.S. Public Health Service Untreated Syphilis Study in Tuskegee, Alabama). These disparities likely affect clinician-patient communication and shared decision-making.

### Implications for practice

Effective clinician-patient communication is critical to improving health outcomes (44). The current study highlights that patients in rural, predominantly African American communities in Alabama often do not receive high-quality communication from providers, signaling a lack of shared decision-making. Addressing this gap requires dual engagement:

• Clinicians must be trained to address biases (e.g., racial, gender, socioeconomic) and prioritize respectful, attentive communication. Using plain language and demonstrating cultural awareness are also key.
• Patients need education and tools to build self-advocacy skills. Programs teaching patients to prepare for appointments, track their treatment experiences, and articulate concerns can enhance communication (45, 46).

Teach-back methods and strategies like Ask Me 3 can also empower patients by clarifying their understanding and reinforcing engagement (47).

### Implications for further research

Future research should include mixed methods to gather both quantitative and qualitative data for a richer understanding of patient experiences. Further exploration of patient-provider gender and racial dynamics is also warranted, particularly the observed disparities in male patient experience.

Additionally, concepts such as “respect” and “listening” require deeper investigation as their definitions may vary from region to region. For example:

• Does “being listened to” mean receiving a desired outcome?
• Does it mean achieving clarity or understanding by the end of a visit?

Research into the use of teach-back techniques and how they correlate with patient perceptions of listening and communication quality would also be valuable.

### Conclusions and educational implications

Improving patient experience necessitates change from both providers and patients. Patients benefit from preparation and active participation during visits, while providers must adopt patient-centered approaches such as shared decision-making. Focused listening, plain language, and cultural sensitivity are essential. Ultimately, patient experience must be prioritized by both parties, and the complexity of healthcare communication must be addressed with empathy and clarity.

### Limitations

There are some limitations to the current study. First, the convenience sample used may not be representative of all rural communities or all rural African American communities. These Alabama communities have a particular history and culture which likely contribute to the patient experience reported. Also, there may be differences in the patient experience as a function of socioeconomic status, race/ethnicity, or gender identity that was not examined. Like many studies, the participants were not balanced on all demographic variables to allow for comparisons. Secondly, we did not obtain information about healthcare providers including their gender, race, and cultural background in order to examine their influence on communication. Finally, there were some limitations to the survey used. The survey has not been validated or tested for reliability. Also, the use of self-reported assessment of the patient experiences via surveys may be subject to memory bias.

## Data Availability

The raw data supporting the conclusions of this article will be made available by the authors, without undue reservation.
