# Supplementary material for: Patient experience during medical visits in predominately African American rural communities in Alabama
Source: Front Health Serv. 2025 Jun 11;5:1420698. doi: 10.3389/frhs.2025.1420698 (PMC12187645; doi:10.3389/frhs.2025.1420698)
Supplement: Supplementary file 1 [file Table1.docx]

Supplementary Materials

**Table S1: Community characteristics**

| **Town** | **Population** | **% African American** | **Per capita income** | **SV Index** |
| --- | --- | --- | --- | --- |
| Community 1 | 1,859 | 85.5% | $19,618 | 0.8283 |
| Community 2 | 2,239 | 88.4% | $14,872 | 0.9537 |
| Community 3 | 1,880 | 35.4% | $22,793 | 0.703 |
| Community 4 | 2,847 | 64.2% | $10,782 | 0.9175 |
| Community 5 | 755 | 90% | $21,439 | 0.8729 |

Note: SV index= social vulnerability index.

Clinician-Patient Communication Satisfaction Survey

Q1 What is your age?

- 18-25
- 26-45
- 46-65
- Over 65

Q2 What town do you live in?____________________________________________________

Q3 What is your Race/Ethnicity? 
(Check all that apply)

- American Indian or Alaska Native
- Asian
- Black or African American
- Hispanic or Latino
- Native Hawaiian or Other Pacific Islander
- White
- Other ________________________________________________

Q4 What is your gender?

- Male
- Female
- Other ________________________________________________

Q5 Was your most recent appointment virtual or in-person?

- Virtual, phone based (audio only)
- Virtual, audio with video
- In person
- Other ________________________________________________

Q6 Have you ever missed an appointment with this clinic/provider?

- No
- Yes

Q7 If you have missed an appointment, why did you miss your previous appointment(s)?
(select all that apply)

- No transportation
- Forgot
- Had to work
- Could not afford it
- Other ________________________________________________

Q8 How would you rate the friendliness/responsiveness of the staff?

- Very poor
- Poor
- OK
- Good
- Excellent

Q9 How long did you have to wait for your appointment to start?

- My appointment started on time
- Less than 30 minutes
- 30 min to 1 hour
- 1 to 2 hours
- More than 2 hours

Q10 Please tell us about your visit:

|  | Never | Sometimes | About half the time | Most of the time | Always |
| --- | --- | --- | --- | --- | --- |
| Did the care providers ask for your opinions? (1) |  |  |  |  |  |
| Did the care providers listen to what you had to say? (2) |  |  |  |  |  |
| Did the care providers treat you with respect? (3) |  |  |  |  |  |
| Was your privacy respected and maintained? (4) |  |  |  |  |  |
| When you asked questions, did you get answers you could understand? (5) |  |  |  |  |  |

Q11 How much information did you get about your condition from your care provider?

- Far too little
- Too little
- About the right amount
- Too much
- Far too much

Q12 Do you know where to go for any new questions after the visit?

- Definitely not
- Probably not
- Probably yes
- Definitely yes

Q13 How satisfied were you with your visit?

- Extremely dissatisfied
- Somewhat dissatisfied
- Neither satisfied nor dissatisfied
- Somewhat satisfied
- Extremely satisfied
